# Engaging Young People With Mental Health Needs and Exploring Outputs From a Resource Development Project: Qualitative Interview Study

**DOI:** 10.2196/74258

**Published:** 2025-08-25

**Authors:** Zoë Haime, Charlotte Carney, Myles-Jay Linton, Helen Bould, Lucy Biddle

**Affiliations:** 1 Population Health Sciences, Medical School University of Bristol Bristol United Kingdom; 2 National Institute for Health and Care Research (NIHR) Biomedical Research Centre University of Bristol Bristol United Kingdom; 3 School of Education University of Bristol Bristol United Kingdom; 4 Centre for Academic Mental Health University of Bristol Bristol United Kingdom; 5 Medical Research Council (MRC) Integrative Epidemiology Unit University of Bristol Bristol United Kingdom; 6 Gloucestershire Health and Care NHS Foundation Trust Gloucestershire United Kingdom; 7 University Hospitals Bristol and Weston NHS Foundation Trust National Institute for Health and Care Research (NIHR) Applied Research Collaboration West Bristol United Kingdom

**Keywords:** qualitative, resource development, children and young people, mental health, research design, participatory research, codevelopment

## Abstract

**Background:**

Recommendations from professional bodies, including the Royal College of Psychiatrists, advise mental health practitioners to discuss problematic online use with children and young people. However, barriers such as knowledge gaps and low confidence in initiating discussions often prevent these conversations from happening.

**Objective:**

The Digital Dialogues project used a knowledge exchange approach, cocreating resources with young people, to support professionals in overcoming these challenges. This paper details the project design and reflects on the perspectives of the young people involved.

**Methods:**

The project was guided by the “children and young people have ownership” model of cocreation. A total of 11 participants were purposively sampled to take part in the Digital Dialogues Young Persons Group (DDYPG) and were actively involved in the study workshops, creative tasks, and resource design and development. In total, 6 (55%) DDYPG members took part in interviews, and 2 (18%) also completed an anonymous survey evaluating their time in the DDYPG. Thematic analysis was used to explore data from interviews and qualitative survey responses together.

**Results:**

The DDYPG successfully created several resources to support practitioners in addressing problematic online use with young people. Reflections from DDYPG members showed that creative engagement, meaningful involvement, and peer interactions were key motivators for participation and led to benefits, including feelings of empowerment and personal development. Anxiety, time demands, and potential exposure to triggering content could act as barriers. However, structured tasks, positive rapport with researchers, and flexible participation helped to mitigate these challenges.

**Conclusions:**

The findings highlight ethical considerations and potential strategies for involving young people in resource development research projects in the future.

## Introduction

### Background

Online use can offer opportunities to children and young people, including learning, connectedness, and fun. In relation to their mental health, it can also encourage access to helpful information and peer support [1,2]. However, there are concerns about the risks associated with online use in children and young people. For instance, links exist between engaging with harmful or distressing images and maladaptive behaviors, including self-harm and disordered eating [3,4]. In addition, negative online experiences have been significantly associated with increased psychiatric symptoms in children and young people [5].

Therefore, recommendations have been made for mental health professionals (MHPs) working with children and young people to support their online use. This includes advice from the Royal College of Psychiatrists [6] for psychiatrists to inquire about online use during all consultations with young people. Research has also shown a willingness among MHPs to discuss this topic with young people [7], but several barriers, including knowledge gaps, time constraints, and a lack of confidence, prevent them from doing so [8,9]. As a result, guidelines have been developed, such as the good practice indicators, which act as advice for those managing these conversations in mental health practice [10]. However, little is known about their implementation in practice, and MHPs continue to lack practical and accessible resources to navigate these conversations effectively [11]; MHPs have expressed a clear interest in tailored training, assessment tools, and evidence-based resources to support their work in this area [11]. Research has highlighted the value of involving children and young people in mental health resource development, showing that such participation can lead to more effective outcomes and promote a sense of empowerment among children and young people [12,13]. At the same time, challenges persist regarding meaningful engagement, with studies emphasizing the importance of nontokenistic involvement and the need for innovative methods of participation [12,13]. Despite these insights, to the best of our knowledge, no project has directly codeveloped practical tools for MHPs to use with children and young people, addressing their online experiences and mental health.

The Digital Dialogues project aimed to use a knowledge exchange approach [14] to develop additional resources for MHPs, aiding their discussions with young people regarding online use. This method was used as it encourages a dialogue between populations, allowing for the integration of both lived experience and professional perspectives and ensuring the resources developed are relevant to both. First, in an evidence-synthesis phase, we conducted 2 nationwide surveys to inquire about (1) what resources and training MHPs want and need [11] and (2) what thoughts and feelings children and young people have about professionals working with them regarding this topic. Second, in a resource development phase, we collaborated with young people, using creative methods, such as art, poetry, and drama, to engage them and allow for self-expression of thoughts and ideas through a variety of means [15]. During this phase, we established the Digital Dialogues Young Persons Group (DDYPG), providing a space for young people with lived and living experience of mental health needs to contribute to Digital Dialogues in member roles.

### This Study

This paper aims to outline and evaluate ways DDYPG members were involved as members in the Digital Dialogues resource development phase. We present details of the workshops, creative tasks, and project processes to demonstrate how we involved and engaged young people, alongside interview data in which participants reflect on their experiences.

## Methods

### Collaborative Approach

We aimed to collaborate with children and young people with lived and living experiences of mental health needs to generate ideas for potential resources based on their experiences and perspectives. Drawing on the *Guidelines for Research with Children and Young People* [16], we focused on approaching the study with the “children and young people have ownership” model of involvement. By doing so, we hoped to provide children and young people with agency over the research process and embed them as research team members while providing guidance and support from the trained research team who helped them navigate [17].

### DDYPG Recruitment

The DDYPG aimed to recruit 8 to 12 young people. A digital recruitment advertisement was distributed via various young people’s groups, including Arts Emergency, Partnership for Young London, and the National Youth Agency, as well as specific mental health organizations, including McPin, OCD Youth, Body Dysmorphic Disorder Foundation, What Works Wellbeing, Mental Movement Magazine, and Beyond. In addition, the advertisement was shared through the Epigram University of Bristol student newspaper and relevant societies at universities across the United Kingdom, including the ThinkMental King’s College London Society, Beat This Together University of Bristol Society, and Student Minds University of York Society.

Potential DDYPG members completed an expression of interest form, detailing their name, email address, age, lived experiences of mental health and online use, and creative interests. They were then assessed against eligibility criteria for involvement (Textbox 1).

After 3 expressions of interest were deemed ineligible, study information sheets were sent to all eligible potential participants (N=45), and of those, 20 (44%) continued to express an interest in participating. After reviewing prospective participants, we selected individuals through purposive sampling and invited them for an individual introductory session with researcher ZH. Purposive sampling was used to ensure a diverse population, prioritizing variation in mental health experiences while also attempting to include a range of demographics and creative interests to enrich the perspectives within the study. During introductory sessions, potential members were able to ask questions, learn about the safety plan and consent process, and provide brief information regarding their online use and mental health experiences.

Following these sessions, the first 11 potential DDYPG members provided consent to take part in the DDYPG, and recruitment was closed as researchers felt confident the group reflected a broad range of relevant experiences. During this process, members gave consent for their contributions to be used and shared in resources and provided separate consent for any potential sharing of their creative work. At this point, they also completed a survey that informed researchers of specific triggers they may have related to mental health content. Recruitment took place over a brief period between October 2023 and November 2023 and was closed once all members had provided informed consent.

**Eligibility criteria.**

- Aged 14 to 25 years
- Lived or living experience of engaging online regarding their own mental health
- Willingness to participate for up to 7 months
- Access to a stable internet connection
- Adequate understanding of the English language
- Currently residing in the United Kingdom

### Ethical Considerations

Ethics approval was given by the Faculty of Health Research Ethics Committee at the University of Bristol (15930). Although this was public engagement work, ethics approval was sought due to the involvement of vulnerable young people with mental health needs, the planned creative outputs, and our intention to evaluate the collaborative work. We wanted to ensure group members were appropriately safeguarded and fully informed about their rights regarding the creation and sharing of materials during the study. Participants provided informed consent on two occasions: initially upon entering the study, and again prior to the creation of resources. The first consent form addressed their involvement as research participants, while the second outlined their rights regarding any intellectual property generated during resource development. Participants were informed that they could withdraw from the study at any time; however, content they contributed to the co-created resources could not be withdrawn. All participant data were handled in accordance with data protection legislation. To ensure confidentiality in this paper, participants have been assigned unique identifiers in place of their names. The young people involved in the study were reimbursed for any time contributed to research or resource development, at a rate of £25 (US $33.23) in vouchers per hour.

As part of our ethical approach, we made it a requirement for the DDYPG members to complete an individual safety plan (Multimedia Appendix 1). In this plan, members provided details of an emergency contact and their general practitioner to be used if researchers identified an immediate risk of harm to themselves or others. In addition, they could create a personalized care plan and access a range of well-being resources. Researchers also followed a distress protocol during the project, including following up with members individually after each workshop.

### Online Platform Communication

As part of this study, we set up a private server on the online communication platform, Discord. The Discord platform supports discussions and has features enabling file sharing. In addition, Discord is a popular platform among young people that has been shown to enhance digital collaboration [18]. We believed this would be an effective way to encourage conversations among young people, engagement with study materials, and sharing of information. While there are other platforms with similar functionalities (eg, Slack and Microsoft Teams), Discord’s widespread use among our target demographic and its intuitive features made it particularly suitable for this study. All DDYPG members and Digital Dialogues researchers were invited to join if they wished, with ZH moderating content. Platform discussions were restricted during nonworking hours.

### DDYPG Procedure

Three DDYPG workshops (Table 1) took place via the online videoconferencing platform Microsoft Teams between November 2023 and January 2024. All workshops were audio recorded, and the audio was transcribed by ZH, who then created and shared a workshop summary with all DDYPG members.

Where young people were unable to attend or preferred not to be involved in workshops, they were given the opportunity to take part in alternative ways, such as involvement in discussions over Discord or creating, revising, and editing documents and resources.

Throughout the project, young people also took part in several creative tasks (refer to the Results section). Instructions for tasks were shared via Discord and email, and for task 1, they were posted to a given address, alongside some creative materials. Creative work was used to encourage young people’s involvement in discussions related to their experiences and encourage idea generation for the resulting resources [20].

After the final workshop in which shared decision-making allowed researchers and DDYPG members to outline what resources the group would create, ZH contacted DDYPG members individually about their involvement. In some cases, members also approached ZH with ideas for resources to develop. Members worked on resources independently or in groups, alongside input from researchers, where indicated as necessary by the young people, between January 2024 and May 2024.

A total of 7 (64%) of the 11 DDYPG members also received training in content analysis methods and contributed to a separate manuscript, and 3 (27%) made content for Digital Dialogues presentations at conferences. In addition, creative outputs by DDYPG members were displayed in a web-based exhibition that members reviewed and provided feedback on.

Following the creation of the resources, Digital Dialogues 2 has been funded, and it commenced in November 2024. This project aims to develop a training package and session for MHPs that incorporates the Digital Dialogues resources. Dissemination of the Digital Dialogues resources is therefore ongoing, with DDYPG members being consulted on an ongoing basis.

**Table 1:** Task aims and instructions.

Task	Aim	Instructions
Task 1: GPI^a^ creative work	Create a visual or written piece reflecting on a GPI [10]	Members received a welcome pack with creative materials and two suggested methods: (1) erasure poems (using pages from books) and (2) smartphone template drawings. They could also use their own artistic style. Creations were shared in workshop 1.
Task 2: survey	Gather young people’s views on online culture, mental health, and digital communication	There was a short survey exploring emoji meanings, mental health platforms, online trends, influencers, and experiences with MHPs^b^. Results were discussed in workshop 2.
Task 3: search history poems	Use list-style poetry to reflect on online searches and mental health experiences	Inspired by poems by Vuong [19] and members created poems using real or fictional search histories to narrate their online journeys. Results were discussed in workshop 3.
Task 4: character development	Develop a character for a mental health video resource	During workshop 3, members brainstormed a “day in the life” concept. They completed character profiles, covering backstory, social media habits, daily experiences, and an MHP interaction (both positive and negative scenarios).

^a^GPI: good practice indicator.

^b^MHP: mental health professional.

### Workshops

DDYPG members took part in 3 online workshops designed to create a space for young people to share their experiences with online use and mental health while also considering the perspectives of MHPs. Before each workshop, DDYPG members received details about preworkshop tasks, what would happen during the workshop, and postworkshop follow-ups (Table S1 in Multimedia Appendix 2 [10,11,19,21-24]). The primary aim of the workshops was to work toward idea generation for resource creation and prepare members to bring their own experiences and insights into the resource development phase.

### Tasks

Tasks were completed to help young people reflect on their personal experiences, with creative methods used to allow novel ways of self-expression. Ultimately, the information gained through workshop discussions of tasks informed the conception and development of resources, ensuring that the perspectives and experiences of all DDYPG members were incorporated. Tasks are described in Table 1, and the details are provided in Table S2 in Multimedia Appendix 2.

### Study Flow

**Figure 1:**
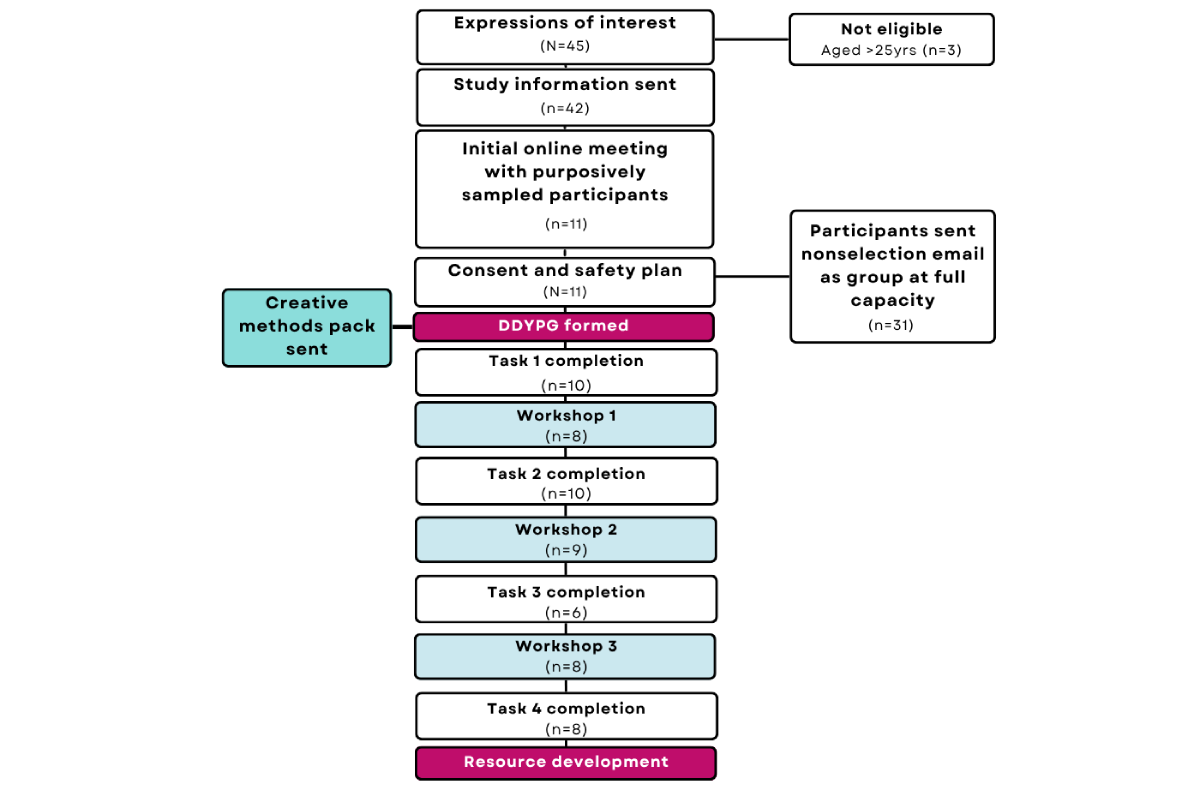
Digital Dialogues Young Persons Group (DDYPG) study participant flow.

The participant study flow is detailed in Figure 1.

### Evaluation of Involvement in Digital Dialogues

All DDYPG members (n=11) were invited to evaluate their time in the study via a one-to-one online interview and by completing an anonymous survey with similar questions, allowing for additional feedback. Interviews were semistructured and conducted by ZH using a topic guide exploring the positives and negatives of involvement in the DDYPG, suggested changes for the DDYPG, reflections on specific workshops and tasks, and opinions on the resulting resources. In addition, all members were invited to complete an anonymous survey, which was designed to provide an additional route for feedback from participants who may have felt less comfortable sharing openly in interviews due to their existing relationships with the researchers. Audio from interviews was transcribed by ZH.

Interview participants are referred to in the results using participant IDs (eg, P01 and P02), while survey responses are labeled with anonymous IDs (eg, anonymous 1).

Researcher CC joined the Digital Dialogues project after the evaluation interviews had been conducted and carried out the initial coding of transcripts using thematic analysis [25]. The involvement of CC ensured a layer of analysis from a researcher not involved with the data collection, helping to enhance rigor and reduce bias. Thematic analysis was chosen for its flexibility and systematic approach. After initial coding, CC then organized the codes and generated themes, which went through an iterative process following feedback from ZH. Coding was conducted in Microsoft Word, and Microsoft Excel was used to organize the data. Then, there was a member-checking phase, where 3 DDYPG members were invited to review the resulting data to ensure accurate representation. This led to minor refinements, such as adding detail to positive changes in online behavior and including more information on flexibility toward member involvement.

## Results

### DDYPG Member Demographics

In total, 8 (72%) of the 11 members identified as female, 2 (18%) as male, and 1 (9%) as nonbinary. All were aged between 18 and 24 years and based in the United Kingdom. Members had experience with a range of mental health difficulties, including anxiety, depression, personality disorders, eating disorders, and obsessive-compulsive disorder.

### Resources

Table 2 presents details of the resources that were conceptualized, designed, and created during this project, with contributions by the DDYPG members given in detail. Resources were created simultaneously, over a period of 5 months, to ensure young people had time to contribute alongside other commitments. Researchers provided feedback and editorial input on resources, which were then reviewed and amended by the group members before a resource was finalized.

**Table 2:** Details of resources developed during the Digital Dialogues project.

Resource	Description	DDYPG^a^ involvement
Flash cards and road maps	These resources included 20 flash cards representing a safety mechanism related to online use, with a lived experience account detailing children’s and young people’s experience of using it; road maps outlined a potential online scenario and the relevant flash cards for MHPs^b^ to use with it.	Three members contributed lived experience content. Five members selected the flash card design. One member conceptualized the creation of road maps and designed them. One member reviewed and edited the road maps. They also reviewed researchers’ changes and made amendments.
Content creator advice poster and additional information	The poster outlined considerations for MHPs when children and young people were following a content creator who posted mental health–related content; a document with more detailed guidance was also created.	One member conceptualized and designed the poster. They also reviewed researcher changes and made amendments. One member developed a detailed information document to complement the poster.
Comfortable conversations with MHP poster and question prompt bank	Poster outlined strategies that MHPs could use to help children and young people feel comfortable during conversations about their online use. The question prompt bank offered MHPs a curated list of important questions to ask children and young people.	Eight members conducted content analysis of a children and young people survey. One member conceptualized and designed the poster. They also reviewed researcher changes and made amendments. One member developed the question prompt bank. They also reviewed researchers’ changes and made amendments.
Video	Video portrayed a “day in the life” of a young person whose behaviors and mood were both positively and negatively impacted by her online engagement, particularly in relation to eating	Eight members produced character descriptions (task 4), which researchers merged to form the lead character. Two reviewed, discussed, and edited the script (made by the research team). One member helped audition potential actors. Eight members provided voice-overs for the video.
Web-based exhibition	The web-based exhibition served as a platform to display the creative outputs produced by DDYPG members throughout the study.	Seven members reviewed the web-based exhibition and gave feedback on changes.

^a^DDYPG: Digital Dialogues Young Persons Group.

^b^MHP: mental health professional.

### Evaluation

A total of 6 (54%) of the 11 DDYPG members completed evaluation interviews, and 2 (18%) took part in the anonymous survey. Results from the thematic analysis of the qualitative response data are presented subsequently by theme.

#### Reflecting on Involvement in Creative Tasks

DDYPG members gave mixed feedback when reflecting on task 1 (Table 2). When approaching the task, one (P03) participant felt completely unable to finish it, and others expressed struggling with it, feeling they lacked the necessary artistic ability:

I’ve never done anything like that before. I guess I am a creative person, but I’m not an arty person, so it was a bit out of my comfort zone.

P01

Another participant (P05) found the task somewhat restrictive due to instructions limiting what content they could focus on, and one delayed the task, which seemed to stem from concerns about how others in the group may perceive their experiences:

I couldn’t decide what I wanted to talk about. At that time, I was aware of my online use and how negative it was when I was younger, there was still a stigma.

P06

However, those who completed the task and presented it during workshop 1, including members who were originally reluctant, named several benefits of involvement. This included giving and receiving positive feedback and discovering new ways to express themselves. A few found the completion of erasure poems during task 1 particularly helpful, as the constraints of the task made it easier to articulate complex thoughts and feelings:

Sometimes with these sensitive topics, if you’ve been through a lot there’s so much to say, that if you’re given text and you have to erase words and work with what you’ve got it forces you to express yourself a certain way…I was expecting it to be difficult because obviously you’re limited, but I thought it was a really good exercise.

P04

In task 3, members were invited to write “search poems” about their online experiences related to mental health. Several also enjoyed the reflective nature of this task:

It wasn’t creating as much as thinking or forcing yourself to reflect on how you use the internet. It’s something you don’t really think about because we use it all the time, but I had to pick out certain things that were common threads for me…it forced me to reflect on the things that I’m actually searching for.

P04

However, one member had a more nuanced reaction. While they recognized the beneficial nature of the reflection, this was balanced with the acknowledgment that revisiting these periods of poor mental health could have had a negative impact if their personal resilience was not as strong:

I think it would depend on someone’s mental state at the time. I can see how that might be slightly triggering, I mean it was quite sad for me to do. It also was a bit of a blur, the period I chose because I was quite unwell, but then it did help clarify that a bit.

P05

In addition, this task was described as “tricky” (P06) due to the challenge of connecting online use with mental health, and P03 struggled with the directions. While fewer members reflected on tasks 2 and 4, P05 found that the character development work (task 4) was “really fun,” and P02 remarked, “I really liked taking part in the survey [task 2] too”.

#### Facilitators of and Barriers to Member Involvement in the DDYPG

We identified several key factors that facilitated as well as posed barriers to successful involvement in the DDYPG.

##### Building Safety and Trust

First, ensuring a safe and trusting environment was integral to DDYPG members’ involvement. Members expressed feeling “it [was] a very safe space, safeguarding was great and was inclusive to all” (anon1), and the requirement to complete a safety plan before involvement reassured members, “it was good to have that precaution” (P06).

Others noted how completing a safety plan would be a “good idea” (P02 and P03) for any mental health research involving young people, and one identified how it helped build rapport between the researcher and the member:

It’s always good to have a safety plan for the young person but also the person doing the research because then at least you have that mutual understanding of what can be helpful and unhelpful during the involvement.

P03

None of the DDYPG members reported needing to access the safety plan during the study. This aligned with their self-perception of being comfortable and “confident” (P06) while discussing sensitive topics.

##### Positive Group Dynamics

The perception of safety was reinforced by the positive group dynamics. Members particularly appreciated the “non-judgemental” attitudes from peers (P01 and P03). The mutual awareness and understanding of handling potentially harmful information also played a role:

Luckily everyone else in the group was probably quite aware of sensitive topics we were discussing and perhaps not going into too much unnecessary detail that might be triggering. So, I’ve never felt super uncomfortable.

P05

An additional factor that influenced DDYPG involvement was the opportunity for members to engage with peers without researchers being present. This facilitated open conversations and allowed for organic idea generation:

I liked how we went into breakout rooms without the researchers, it felt like we were just talking young person to young person. Although the researchers are here and they understand the topic and they want to make a difference and make a change, a lot of the time they won’t have had these experiences before, sometimes that can make it difficult to talk to them.

P06

Members also emphasized the value of contributing to research that could help others. This sense of purpose encouraged their involvement and made them feel connected to a group of like-minded individuals:

It was good to talk to young people who want to be involved in a project to make a difference and therefore are happy to talk about and share their experiences.

P06

##### Valued Members of the Project Team

DDYPG members consistently mentioned the quality of their involvement in the project as a significant motivator. The supportive relationships with researchers were a key factor, “I felt very cared for and valued.” (anon2), and researcher responsiveness also played a role:

There were times where I would send you these huge rants in emails of all this stuff I noticed online, and you [the researcher] made sure I felt validated and I felt heard, which is really important for me.

P02

In addition, the high level of DDYPG involvement in the study process was crucial. One participant remarked as follows:

I feel like we’ve genuinely been quite equal partners in all of it, which is really cool.

P01

This level of involvement was directly compared to other cocreation roles the group had been involved in:

[The DDYPG] were different to other young people’s co-creation, they had a variety of different methods and options to choose from. I didn’t feel limited in any way.

anonymous 1

This promoted a sense of empowerment and encouraged the DDYPG members to question their other cocreation roles:

The level of involvement we’ve had has made me challenge a little bit [in other cocreation roles], like, “Why can’t we have more involvement? Why can’t we be doing this? Why can’t we be involved in that?”

P01

##### Time Management

Scheduling flexibility also facilitated involvement in this project and individual tasks. Members generally felt that their commitment to the DDYPG was “manageable” (P01), as “involvement was fairly spaced out” (P03), and they could “balance” (P04) it with other work, including university and jobs. In addition, the ability to continue conversations about task work on the Discord platform facilitated this flexibility:

I appreciated the opportunity to participate in the tasks but then not necessarily have to be in the meetings to have discussions because they could move to Discord...I felt like we had good opportunities to participate in various different ways.

P01

A few members also noted that if tasks had shorter deadlines or were set all at once, it may have been “overwhelming” (P03) and could have hindered their involvement. However, one member (P04) did feel they missed out on some involvement due to university obligations.

A couple of members observed that it was their responsibility to manage time and assess their capacity to complete DDYPG tasks alongside their daily lives. One recalled declining involvement comfortably:

There was a time I remember where you sent two tasks, and I was like to be completely honest I only really have time to do one, and you were like that’s absolutely fine just do the one. That was quite nice, as much as I wanted to do the other one, I had to be realistic, you know, I’ve got a bunch of exams coming up, I don’t know if I can do both of those.

P02

However, the other participant felt less comfortable rejecting tasks, though they appreciated that presenting them as optional made decision-making less pressured:

You gave me the option, “would you like to do this” rather than “we’re going to do this,” I felt more able to say no. Although I never said no because I liked the project and I wanted to be involved, but I did like that I had the opportunity to say no or later on down the line I could be like “I don’t have time to do this, I’m sorry.”

P06

##### Anxiety

Most members expressed initial anxiety about attending the first DDYPG meeting, which could have acted as a barrier to involvement. While 2 participants (P02 and P03) attributed their apprehension to social anxiety diagnoses, others (P01, P03, P05, anon1, and P06) shared similar concerns. They mentioned unfamiliarity with group members, fears that conversations might be triggering, and anxiety about presenting their creative work, especially when comparing it to the unknown pieces others had produced. However, all of these members also mentioned that the anxiety quickly dissipated once the first meeting began:

I’d say just the nervousness of going on a zoom call with loads of people I don’t know and wondering if it’s going to be triggering or if it’s going to have an impact. And the nervousness of taking a piece of art and wondering what is this going to look like compared to everyone else...but I think that disappeared within five minutes of being on the call because everyone was just so nice and it was great to get to know everyone a little bit.

P01

Another concern that was mentioned by a couple of members was that their experiences would not align with the group’s “norm” regarding mental health and online use:

What if my idea about being chronically online and how harmful it is isn’t the norm?

P06

In addition, one member expressed concern that their perspective might be “a really bad representation of people’s experiences” (P05). This worry persisted throughout the project, as they explained the following:

It was in the back of my head that I didn’t want to say something—not wrong, but different or not representative enough.

P05

#### Member involvement in the DDYPG: Benefits and Risks

##### Validating Experiences

One of the primary benefits identified by DDYPG members was the opportunity to engage with individuals who had similar stories to theirs. This was seen as a chance to honestly talk about their mental health and online use (“I felt positive about being able to share my experiences” [anonymous 2]) and hear from others, which many described as “validating” (P01, P02, P04, and P06). One communicated how this shared understanding helped them gain deeper insights into their own experiences:

I really enjoyed seeing the perspectives of people who’d been in similar situations to me and that helped me understand that side of using the internet in relation to my mental health a bit more.

P04

Young people also appreciated the chance to engage with peers who may have had different experiences from them, finding it valuable and “interesting” (P04) to “[learn] more about other people’s perspectives” (P01). This not only broadened their understanding of mental health but also helped them challenge their own preconceptions:

it was cool to know more things about them [DDYPG members’ mental health conditions], and probably addressing some of my own assumptions about them too...

P05

##### Positive Change in Online Behaviors and Mental Health

In addition, involvement in the DDYPG led some members to reflect on and adjust their own behaviors to become more deliberate with how they navigated the online world, such as by spending less time online or changing the content they engaged with. For instance, one member stated the following:

I became more reflective about how I use my time online. I’m someone who likes to do scrolling like everyone else, so it felt a bit more intentional.

P02

Another noted that they started to critically evaluate other online users, which impacted their time spent online:

I noticed in my [online] use as well, that person is doing that that doesn’t make them a very good influencer, so thinking about this [research] was impacting my use too.

P06

Some of the members also reflected on the potential “therapeutic” (P01) value of involvement in Digital Dialogues, specifically in the creative tasks. One shared how writing the “search poem” allowed them to access and reconnect with their mental state during a difficult time, which had an overall positive impact:

My poem was about self-harming, and I think about it from my perspective now quite logically but my poem was that voice from when I was going through it. That made me connect to that situation more. I went back to how I was feeling rather than trying to intellectualise it...Just going back to how I felt and what it meant and why it happened, that was difficult but quite therapeutic and overall positive.

P04

Another member, who had some previous experience using creative methods to support their mental health, valued the option to explore a new outlet:

I’ve never really written poetry it was kind of therapeutic and I have now considered it.

P05

One participant started using poetry as a therapeutic tool as a direct result of their involvement:

I tend to write poetry now...Sometimes it’s around online use and sometimes generally mental health but I hadn’t thought about using creative outlets like poems until after I’d started in the Digital Dialogues project.

P06

##### Personal Development Opportunities

DDYPG members also highlighted how their involvement in Digital Dialogues positively impacted their individual development. One noted that being listened to and seeing their contributions being used gave them self-assurance:

I think it helped my confidence quite a lot. Like I’ve said, knowing that my opinions were being heard and valued and they weren’t just thoughts I have that would fall on deaf ears and would never really make a change or anything.

P02

A participant also felt valued during the study and had pride in their role:

Being involved has allowed me to feel like I’ve had a sense of purpose and more fulfilment in life. It’s helped with my general mood and feeling like I’m actually trying to make a difference. That’s the main thing that’s been positive, just that sort of feeling that I’m doing something that’s productive.

P03

Interestingly, 3 DDYPG members also mentioned how involvement in the project helped them overcome internalized stigma, which had previously stopped them from talking openly about their mental health and online use:

It was difficult talking to people [in the group] originally about my experiences because I’d had this negative experience [talking to friends] in the past. But that was cleared up as soon as people started talking and I was like it’s not just a me thing, other people have experienced this and I’m not alone in this situation.

P08

##### Triggering Effect of Conversations

Members identified that being in this project could also involve risks, including them being triggered by mental health–related discussions. One participant shared that involvement in the study heightened their awareness of the online world, which left them more inclined to occasionally attend to potentially harmful content:

I guess on the negative side, particularly things about suicide these things are darker and deeper than it may appear to be, so when you notice that it can make you feel a bit sad.

P02

In addition, one participant reflected on the potential negative impact that discussions about specific platforms or content could have, noting that this may be dependent on their stage of recovery:

I would still say I’m recovering from an eating disorder so to be given a list of like “so I had difficulty with these specific forums or these websites,” if I was worse, I probably would have looked them up. You have no way of knowing with all the other participants what level of recovery they’re at and if they might use that as a source...I definitely think it could have potentially done that for some people...I think there’s definitely a risk there.

P05

This member suggested researchers “ask people to explicitly avoid naming websites” to avoid these triggers during conversations.

One participant also acknowledged that comparison to other members and triggering content were inherent risks in such discussions but felt these were managed well in the project through the use of content warnings and the availability of researchers:

There were aspects of that that were a bit like oh okay this doesn’t feel quite so nice, and I think that’s always a potential when working with other people with that comparison and that triggering element. But I think overall, that was managed really well in terms of having trigger and content warnings and researchers in the meeting to talk to separately. So, I don’t think it’s had any negative impacts on me.

P01

#### Young Persons’ Reflections on Resource Development

##### Thoughts on the Resource Development Process

The resource development period was viewed positively by members, such as P02, “I think I most enjoyed creating the resources,” and anon1*,* “it had a great positive impact, I felt included, heard and seen.” Before beginning this part of the project, members were asked to identify the types of resources they would be interested in working on. Following this information, ZH approached members of the DDYPG to contribute either together or individually to the different stages of resource development. Members of the group who worked on specific tasks shared some reflections.

P05 described developing the video script alongside P01, highlighting how they were able to bring their own lived experience to the work and felt free to give honest input on the existing script. They appreciated the collaborative atmosphere, where they could engage critically while also sharing moments of humor related to their online use and mental health:

It was good to do the scriptwriting with [P01] too, that was really interesting. I enjoyed the conversation because it was funny and we could have a laugh, but also, we were able to be quite critical of the script. Again, some of my ideas were probably quite different to her and that reflects how everyone’s experiences are very different.

P05

One participant also reflected on this collaborative relationship, noting the value of both being able to contribute their own perspectives:

The fact [P05] did the video script with me, I think it was really nice that we were the ones who had that kind of experience so we got to do the scriptwriting.

P01

A participant also described their involvement in the actor audition process, noting, “[I found] auditioning the actor really fun, I’ve never had to audition someone before, and I really enjoyed that actually” (P05). This involvement also prompted a deeper reflection on the representation of mental health in resources, such as those we created:

The last thing I wanted to do [while writing the script] was stereotype. I think that’s why it was important I was there for the auditions because I think some candidates erred on that side of it becoming a bit of a caricature, which we didn’t really want, and it also helped me think a bit more critically about portrayals of mental health.

P05

One participant also reflected on their individual role in designing and developing a poster and question bank directed at MHPs having comfortable conversations with young people about their mental health and online use. They noted that this was not an easy process for them due to concerns that it would not be what the group hoped for:

The question bank as well. To me the question bank was really important, which is why it took me so long to do, I procrastinated on it for so long because I felt like it needed to be perfect.

P06

In addition, members generally reported their appreciation for the diverse roles they were able to have during resource development:

I think it was great to give young people choice and options to co-create through a variety of means and at a time and pace that works for them.

anonymous 1

##### Children’s and Young People’s Perception of Resource Use by MHPs

In total, 5 members expressed hopes that the resources created would provide an opportunity to improve the experience of children and young people accessing support from MHPs. A participant reflected on the potential for MHPs to use them as communication aids, facilitating conversations:

I hope it’ll build communication and help MHPs to be a bit more comforting with the language that is used and the questions that are asked. I’m hoping it’ll be a good way to educate MHPs on how they can support younger people, as that’s the main aim of it, and hopefully the outcome.

P03

Another expressed similar hopes, suggesting that resources could provide practitioners, specifically those working in children and adolescent mental health services, with a “different lens” through which they could understand and talk to young people about online use:

I’m hoping these will be a great prompt for people to take into their own practice and use to make young people feel more comfortable and not judged, because ultimately that’ll be the difference between them engaging with you and completely not.

P05

However, one member noted that the plans for disseminating resources to professionals were unclear to them, which meant they were uncertain about the potential impact:

I guess I wasn’t entirely sure what the plans were in terms of how you send them out. As in, is it every mental health professional, how is that possible? How do you even begin a task like that? That’s the only slightly grey area that once we’ve made these things, I wasn’t entirely sure how they would then get sent to people.

P02

## Discussion

### Principal Findings

Using creative methods, the Digital Dialogues project engaged young people in a research group where they shared their experiences and perspectives on online use and mental health. These discussions resulted in the iterative development of resources for practitioners, a web-based exhibition of creative works, and additional outputs. Findings from interviews with DDYPG members revealed that key motivators for participation included creative engagement, quality of involvement, and peer interaction, which contributed to perceived benefits such as personal development, empowerment, and positive therapeutic outcomes. However, anxiety and time demands were identified as potential barriers to involvement, along with risks, such as exposure to triggering or harmful content. Notably, the data also provide some evidence of steps that helped mitigate these barriers, allowing us to highlight key ethical considerations and potential strategies for future resource development projects.

### The Creative Process

Creative methods enabled young people to articulate their perspectives on online use and mental health within the group. This approach became an effective way to explore complex emotions and experiences [20]. Generally, children and young people appreciated the novel approach, with high levels of task engagement. This was consistent with research showing that creative methods can enhance research involvement by offering alternative forms of expression [26]. DDYPG members also described how they particularly valued constraint-based creative tasks, such as poetry writing. Here, structure helped them describe their personal stories and communicate emotions that might otherwise remain intangible [27]. In addition, young people reported that reflective aspects of creative tasks led to positive changes in online behaviors. This reflects findings from the Delve study [27] where increased metacognitive skills led to positive behavioral changes online.

However, several DDYPG members also reported initial anxiety regarding producing or sharing their creative work, reflecting what Hochman and Esteves [28] term “art fear.” This also echoed observations by Novak-Leonard and Robinson [29] that individuals with limited perceptions of themselves as artists are less likely to engage in arts-based activities. To address this challenge, we adopted several strategies that had a positive impact on facilitating involvement and reducing negative emotions regarding the creative tasks. First, we gave members flexibility by allowing them to use their preferred creative method to complete task 1. This gave them the opportunity to draw on their strengths and work in a familiar way, using an assets-based approach [30]. Similarly, tasks were framed as reflective rather than evaluative, aiming to mitigate performance-related anxiety [31]. Finally, we modeled involvement by having researchers share their own work first, a practice highlighted by Leavy [32] as effective in normalizing creative engagement and reducing power imbalances.

### Relationships Within the Research Team

A key facilitating factor for member involvement included the positive peer relationships they built and were able to rely on during the project. This led to feelings of safety, recognition, and validation among the young people, which may have enhanced engagement and confidence [12]. This was further reflected through members’ appreciation for opportunities to work independently with their peers. This movement away from researcher-led formats of collaboration better recognizes the competency of children and young people and may also improve their commitment to the research [33]. However, although young people were able to work and contribute individually to resources, some hesitated to share their input due to concerns about not meeting group expectations or fear of being judged. This anxiety could sometimes delay contributions and may have led members to withhold valuable ideas. Such challenges have been reported in previous research [34].

Rapport between DDYPG members and researchers was also integral to the young people’s active involvement in this project. Members reported that researchers were approachable and responsive, facilitating ongoing discussion, taking them seriously, and making them feel safe. This highlights the importance of researchers having the skills necessary to effectively collaborate with young people in their research, demonstrating a genuine commitment to authentic engagement, addressing power imbalances, and dedicating time to meaningful interactions [17].

### Ways of Working

Members also valued the responsibility and trust placed on them in their roles on this project as well as the flexibility to contribute through various means. Other studies have also shown that offering several options for involvement in engagement work can improve inclusion [35], and using online communication platforms, such as Discord, can enhance this collaboration [18]. In addition, members reported feelings of empowerment comparable to those experienced by others who have participated in meaningful coproduction projects [36]. Furthermore, our members highlighted flaws in other collaborative roles they had undertaken, revealing how effective collaboration can inspire critical reflection on past experiences and empower children and young people to challenge insufficient involvement.

Our working approach adhered closely to the principles outlined in the *Guidelines for Research with Children and Young People* [16]. Specifically, the development phase followed the “children and young people have ownership of the research” model, which emphasized providing children and young people with as much agency as possible. This approach empowered members, giving them a sense of fulfillment in their role. However, despite researchers’ efforts to maintain a manageable workload for children and young people, one individual reported occasionally taking on more work than they could accommodate, driven by their enthusiasm for the study and desire to contribute. This emphasizes the need for researchers to continually balance giving children and young people agency with protecting their well-being [33].

### Managing Sensitive Content Discussions

DDYPG members appreciated how peers were mindful during discussions to avoid triggering content, interpreting this as a skillful use of boundaries grounded in a shared understanding of mental health challenges. In addition, they recognized that their own stage of recovery was likely a key factor in their ability to cope with the discussions and tasks. This may reflect our group composition, as the recruitment strategy targeted children and young people within mental health organizations or groups, where previous experiences may have helped them develop skills in navigating boundaries, addressing sensitive topics, and working collaboratively. It could also reflect the influence of group rules introduced and discussed during the initial workshop.

However, some members noted that despite efforts to avoid triggering content, information that could be potentially harmful was still shared. This suggests that the nature of conversations about mental health and online use may inherently involve exploring difficult or potentially triggering topics, which presents a challenge for researchers in balancing open dialogue and the emotional safety of children and young people. Considering participants’ recovery stage during recruitment may therefore be an important factor. Research shows that those with lived experience of mental health conditions experience varying levels of hope (meaning confidence and symptoms) at different stages of recovery, likely meaning they are able to contribute and cope to varying extents in research roles [37].

In an attempt to overcome potential risks of triggering content in this study, we provided opportunities for members to take breaks and access researchers for support during discussions in separate web-based breakout rooms and ensured postmeeting check-ins. However, it remains unclear whether there may be longer-term negative or positive effects on children’s and young people’s well-being or behaviors because of their involvement in research of this nature.

### Adhering to Perceived Norms

Some members expressed concerns about accurately representing their mental health experiences, reporting a perceived pressure to conform to a “norm” associated with their diagnosis. Notably, this conformity to align with a mental health identity has recently been observed in individuals using social media, where online moderation and in-group formation play key roles in reinforcing diagnostic “norms,” particularly among young people [38,39]. These findings also reflect broader concerns with research engagement, such as the influence of Western societal expectations and desirability biases on participants’ willingness to engage in honest disclosures during mental health discussions [40]. In addition, our efforts to minimize harm by introducing rules to avoid discussing triggering content may have created pressure for members to conform to a sanitized narrative.

Therefore, the inclusion of children and young people with diverse mental health conditions had the potential to create a dynamic where individuals with less common diagnoses felt pressure to represent their condition. However, while this was an anticipated concern among members, it did not appear to be an influence once they took part in tasks and workshops. The group diversity also provided benefits, offering valuable peer learning opportunities and contributing to a potentially destigmatizing environment. This supports research suggesting that diversity in groups can encourage broader perspectives and reduce stigma by exposing individuals to varied lived experiences [41]. Similarly, such diversity may enhance the generalizability of research insights by incorporating a wider range of perspectives.

### Perceptions of Project Outcomes

Members gained confidence from their involvement in this study, reflecting the concept that seeing ideas transformed into practical and tangible outcomes is empowering [42]. They took pride in the resources created and felt hope that they would have an impact on MHPs, improving the ways they speak to children and young people about their online use and mental health. However, members noted gaps in their understanding of how we planned to disseminate the resources to MHPs, a feature previously highlighted as important in collaborative research with young people [43].

### Limitations

This project successfully engaged young people as active contributors to the research through open discussions and creative work. DDYPG members played a key role in developing several resources for MHPs. However, the limitations mentioned subsequently highlight areas for reflection and potential improvements in resource development work with young people.

Members in this study generally reported a willingness to talk about their mental health with others, which made open and constructive discussions possible within the group. However, this also highlighted a potential self-selection bias in this type of research where those more comfortable discussing sensitive topics are more likely to be involved, and individuals who are less inclined to talk about their experiences may be underrepresented [17]. We tried to overcome this limitation by allowing children and young people to be involved in the study in a variety of ways, including through an online discussion platform (Discord) and by commenting on and editing documents.

In addition, one member noted uncertainty about the process for disseminating the resources to MHPs. While this was a general limitation of the project, due to the need for additional funding to support this stage, it is important to consider that the lack of a clear dissemination plan from the outset may have reduced children’s and young people’s sense of ownership or purpose in relation to the resources.

Finally, not all members participated in the evaluation interviews or the anonymous survey. Due to the anonymity of the survey, we cannot confirm whether those who completed it differed from those who took part in the interviews. As a result, we may have missed valuable perspectives from some members that could have provided additional insights.

### Future Directions

This study enabled the creation of several freely available resources for mental health practitioners, hosted on the Digital Dialogues website [44]. It has also informed the Digital Dialogues 2 project, an ongoing research study codeveloping a training package and toolkit in collaboration with MHPs, into which several of these resources will be incorporated. To build on this work and address limitations identified in this study, ongoing dissemination efforts are needed to ensure that these resources are being used meaningfully in practice. As part of Digital Dialogues 2, we will begin exploring how the co-created resources are used and experienced in practice by evaluating the experiences of MHPs who attend our pilot training. Future knowledge exchange projects in this field should continue to prioritize the coproduction and creative methodologies highlighted in this study.

### Conclusion

Involving young people with lived and living experience of mental health difficulties as research team members in a resource development project can be mutually beneficial for researchers and members. Using a structured format of workshops and creative tasks can encourage active involvement and result in collaboratively conceptualized and designed resources being created, with an enhanced level of authenticity. According to DDYPG members, their role in this project was associated with positive outcomes, including empowerment, improved mental health, and a sense of validation. To enable this, it was important that researchers created a safe space and encouraged children’s and young people’s agency and ownership over project decisions. However, challenges remained, including exposure to potentially triggering content, the fear of judgment, anxiety about participation, and concerns about the impact of the developed resources. Through this evaluation, we have identified several mechanisms, as highlighted by children and young people, to navigate and overcome some of these difficulties.

## Appendix

Multimedia Appendix 1 Safety plan template.

Multimedia Appendix 2 Workshop and task design.

## Abbreviations

DDYPG: Digital Dialogues Young Persons Group
MHP: mental health professional

## Data Availability

Data generated during this study are not publicly available due to the sensitive nature of participant contributions and original intellectual property generation, as agreed with University of Bristol data protection services. However, anonymized interview transcripts and qualitative survey response data are available from the corresponding author on reasonable request.

## References

[1] Pretorius C, Chambers D, Cowan B, Coyle D (2019). Young people seeking help online for mental health: cross-sectional survey study. JMIR Ment Health.

[2] Charmaraman L, Sode O, Bickham D (2020). Adolescent mental health challenges in the digital world. Technology and Adolescent Health: In Schools and Beyond.

[3] Dane A, Bhatia K (2023). The social media diet: a scoping review to investigate the association between social media, body image and eating disorders amongst young people. PLOS Glob Public Health.

[4] Jacob N, Evans R, Scourfield J (2017). The influence of online images on self-harm: a qualitative study of young people aged 16-24. J Adolesc.

[5] Weigle PE, Perry K, Kaliebe KE (2023). How online experiences impact adolescent mental illness and what to do about it. J Am Acad Child Adolesc Psychiatry.

[6] (2020). Technology use and the mental health of children and young people. Royal College of Psychiatrists.

[7] Clarke AM, Chambers D, Barry MM (2017). Bridging the digital disconnect: exploring the views of professionals on using technology to promote young people's mental health. Sch Psychol Int.

[8] Derges J, Bould H, Gooberman-Hill R, Moran P, Linton MJ, Rifkin-Zybutz R, Biddle L (2023). Mental health practitioners' and young people's experiences of talking about social media during mental health consultations: qualitative focus group and interview study. JMIR Form Res.

[9] Rifkin-Zybutz R, Turner N, Derges J, Bould H, Sedgewick F, Gooberman-Hill R, Linton MJ, Moran P, Biddle L (2023). Digital technology use and mental health consultations: survey of the views and experiences of clinicians and young people. JMIR Ment Health.

[10] Biddle L, Rifkin-Zybutz R, Derges J, Turner N, Bould H, Sedgewick F, Gooberman-Hill R, Moran P, Linton MJ (2022). Developing good practice indicators to assist mental health practitioners to converse with young people about their online activities and impact on mental health: a two-panel mixed-methods Delphi study. BMC Psychiatry.

[11] Haime Z, Griffiths G, Linton MJ, Bould H, Biddle L (2024). Mental health practitioners training needs and preferences for addressing online use with children and young people. Evid Based Pract Child Adolesc Ment Health.

[12] Mawn L, Welsh P, Stain HJ, Windebank P (2015). Youth Speak: increasing engagement of young people in mental health research. J Ment Health.

[13] McLaughlin H (2006). Involving young service users as co-researchers: possibilities, benefits and costs. Br J Soc Work.

[14] Doing more with what you know: a toolkit on knowledge exchange. Provincial Centre of Excellence for Child and Youth Mental Health.

[15] Rouncefield-Swales A, Harris J, Carter B, Bray L, Bewley T, Martin R (2021). Children and young people's contributions to public involvement and engagement activities in health-related research: a scoping review. PLoS One.

[16] Shaw C, Brady LM, Davey C (2011). Guidelines for research with children and young people. National Children’s Bureau.

[17] Biddle L, Haime Z (2024). Realising the potential of participatory research in youth mental health: time to go back to basics. JCPP Adv.

[18] Banson J, Hardin CD (2022). Assessing student participation and engagement using discord. Proceedings of the IEEE 46th Annual Computers, Software, and Applications Conference.

[19] Vuong O (2022). Amazon history of a former nail salon worker. Time Is a Mother.

[20] Robinson Y, Gillies V (2012). Introduction: developing creative methods with children and young people. Int J Soc Res Methodol.

[21] Marsh H (2014). Do No Harm: Stories of Life, Death and Brain Surgery.

[22] O'Sullivan S (2016). It's All in Your Head: Stories from the Frontline of Psychosomatic Illness.

[23] Gómez M (2022). Engaging with historically marginalized voices through blackout poetry. Soc Stud Res Pract.

[24] Choe NS (2023). Understanding the value of art prompts in an online narrative medicine workshop: an exploratory-descriptive focus group study. Med Humanit.

[25] Braun V, Clarke V (2021). Thematic Analysis: A Practical Guide.

[26] Phillips OR, Harries C, Leonardi-Bee J, Knight H, Sherar LB, Varela-Mato V, Morling JR (2024). What are the strengths and limitations to utilising creative methods in public and patient involvement in health and social care research? A qualitative systematic review. Res Involv Engagem.

[27] Kiernan F (2020). Emotion as creative practice: linking creativity and wellbeing through the history and sociology of emotion. Int J Wellbeing.

[28] Hochman A, Esteves KJ (2021). Art and exceptionality: addressing art fear and fear of difference in an introductory art course. Can Rev Art Educ.

[29] Novak-Leonard JL, Robinson M (2020). Initial findings from a national survey of self‐perceptions of creativity. National Endowment for the Arts Research Labs.

[30] Elsen F, Ord J (2023). ‘You don’t get ditched’—young people’s mental health and youth work: challenging dominant perspectives. Youth.

[31] Bullock Muir A, Tribe B, Forster S (2024). Creativity on tap? The effect of creativity anxiety under evaluative pressure. Creat Res J.

[32] Leavy P (2020). Method Meets Art: Arts-Based Research Practice.

[33] Cuevas-Parra P (2021). Deconstructing the role of adult facilitators in research led by young people. J Youth Dev.

[34] Boswell N, Woods K (2021). Facilitators and barriers of co-production of services with children and young people within education, health and care services. Educ Child Psychol.

[35] PARTNERS2 writing collective (2020). Exploring patient and public involvement (PPI) and co-production approaches in mental health research: learning from the PARTNERS2 research programme. Res Involv Engagem.

[36] Mayer C, McKenzie K (2017). '…It shows that there's no limits': the psychological impact of co-production for experts by experience working in youth mental health. Health Soc Care Community.

[37] Copic V, Deane FP, Crowe TP, Oades LG (2015). Hope, meaning and responsibility across stages of recovery for individuals living with an enduring mental illness. Aust J Rehabil Couns.

[38] Feuston JL, Taylor AS, Piper AM (2020). Conformity of eating disorders through content moderation. Proc ACM Hum Comput Interact.

[39] Corzine A, Roy A (2024). Inside the black mirror: current perspectives on the role of social media in mental illness self-diagnosis. Discov Psychol.

[40] Bergen N, Labonté R (2020). "Everything is perfect, and we have no problems": detecting and limiting social desirability bias in qualitative research. Qual Health Res.

[41] Corrigan PW, Rüsch N, Scior K (2018). Adapting disclosure programs to reduce the stigma of mental illness. Psychiatr Serv.

[42] Culpin I, Dermott E, Ives J, MacLeavy J (2021). Tangible co-production? Engaging and creating with fathers. Area (Oxf).

[43] Bailey K, Allemang B, Vandermorris A, Munce S, Cleverley K, Chisholm C, Cohen E, Davidson C, El Galad A, Leibovich D, Lowthian T, Pillainayagam J, Ramesh H, Samson A, Senthilnathan V, Siska P, Snider M, Toulany A (2024). Benefits, barriers and recommendations for youth engagement in health research: combining evidence-based and youth perspectives. Res Involv Engagem.

[44] Digital dialogues homepage. Digital Dialogues.

