# Supplementary material for: Engaging Young People With Mental Health Needs and Exploring Outputs From a Resource Development Project: Qualitative Interview Study
Source: J Particip Med. 2025 Aug 25;17:e74258. doi: 10.2196/74258 (PMC12417902; doi:10.2196/74258)
Supplement: Multimedia Appendix 1 [file jopm_v17i1e74258_app1.pdf]

My Safety Plan

Digital Dialogues Study

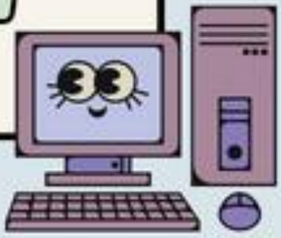

What's your name?	
What's your email address?	
What's your phone number?	

We will only contact your GP and/or safety contact when we believe there is an immediate serious risk to you, or others.

What's the name of your GP surgery?	
What's the address of your GP surgery?	

My **safety contact** is called

They are my

You can contact them at

Self Care

Digital Dialogues Study

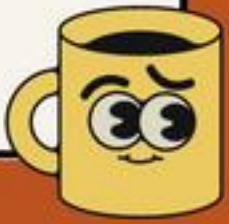

Activities I like to do!

(e.g., read a book, draw, have a cup of tea, go to the gym)

Safe or favourite foods/drinks?

Therapeutic Strategies?

Places I like to be!

(e.g., my room, the beach, my favourite café)

Ways I like to socialise or get support!

(e.g., with my pets, online, from my therapist)

Sleep strategies?

Resources

Digital Dialogues Study

SAMARITANS

A 24-hour service providing confidential emotional support to anyone in crisis.

www.samaritans.org

CAMPAIGN AGAINST LIVING MISERABLY CALM

Charity with a free, confidential and anonymous helpline & webchat service offering help to anyone in a crisis.

www.thecalmzone.net

Beat Eating disorders

Charity supporting those affected by eating disorders.

www.beateating disorders.org.uk

mind

A mental health charity offering advice and information.

www.mind.org.uk

GLITCH

Charity aiming to end online abuse by educating people on how to positively engage in digital spaces.

www.glitchcharity.co.uk

shout
for support in a crisis

Free and confidential, 24-hour text messaging support service for anyone struggling to cope.

www. giveusashout.org

If you feel in need of immediate support, please contact NHS Choices (www.nhs.uk/111) on 111 (available 24 hours a day, 365 days a year, and free). Alternatively, please go to, or call, your nearest accident and emergency (A&E) department and tell the staff how you are feeling.

If you need to speak to a member of the Digital Dialogues research team about your involvement, please feel free to contact Zoë at zoe.haime@bristol.ac.uk
