# Supplementary material for: Engaging Young People With Mental Health Needs and Exploring Outputs From a Resource Development Project: Qualitative Interview Study
Source: J Particip Med. 2025 Aug 25;17:e74258. doi: 10.2196/74258 (PMC12417902; doi:10.2196/74258)
Supplement: Multimedia Appendix 2 [file jopm_v17i1e74258_app2.docx]

**Table A. Workshop titles, and information shown to DDYPG members prior to workshops for activities before, during and after the workshop.**

| **Workshop** | **Before** | **During** | **After** |
| --- | --- | --- | --- |
| **1. Introductions and GPI Task Discussion** | - Group members will take part in the 'Good Practice Indicator' creative task (sent to you individually) and send through their creations to ZH, if they are happy to share. - Group members will read-through the 'Group Ground Rules' and 'Inclusivity' documents. If you have immediate thoughts about these, or need to request adjustments before attending a workshop, do just let us know in advance. | - We will have a brief round of introductions so we know who everyone is. - Group Ground Rules will be established within the group through discussion and amendments to the existing document. We will also have room to discuss any thoughts you have about our inclusivity criteria and you will have the opportunity to amend this. - Thoughts on involvement in the 'Good Practice Indicators' creative task will be shared. As well as what you produced, if you are willing to share (ZH and LB will present first). This will lead into a discussion about what you would prioritise in conversations with mental health professionals on this topic. | - The group meeting will be transcribed and a summary of ideas/discussions will be available to all. - We will decide the best format for the next session, in which we will present previous study findings. |
| **2. Research Background** | Group members can take part in the short survey, if you wish to do so. | - ZH and LB will spend the session going over some findings about what our mental health practitioners already know, and what training and development they still need. As a group we will have a think about what we know in relation to what MHPs want to learn.   Use Padlet to aid discussions. | - We will begin to think about what resources we want to create, which will be discussed in the next session. - Group members will receive an optional additional task [Search History Poem] to participate in. |
| **3. Search Poem Task and Digital Development Discussion** | Group members can complete the poetry task that has been emailed to you if you wish to do so. You can read the two poems attached to gain insight into the structure and formatting of these poems.  Please take a look at the project website [www.digitaldialogues.co.uk](http://www.digitaldialogues.co.ukm)  Think about what resources you might want to be involved in making for mental health professionals. | You will spend some time at the start of the meeting discussing the poems you have created before the session, via breakout rooms in small groups with your peers. We will come back together to highlight and discuss any notable content that may help our work with MHPs, such as any patterns we see in online journeys related to mental health.  We will begin to think of ways we can turn our current ideas and knowledge into resources, thinking about what we want to make and how this may work. We will also briefly discuss ways we might want to share our creative work output at the end of the study.  The Digital Education Developer will introduce himself and his work in this area. He will present some ideas for potential resource work and as a group we will discuss what this might look like, and what content it could include. | If undecided during the meeting, we will vote for the top resources we want to collaboratively create.  We will ask people to make us aware of what roles they would be happy to undertake during the resource creation process.  If we want to pursue a video or animation, you will asked to complete the character design task, if you wish to do so. |

**Table B**. **Task Design**

| **Task Aim** | **Task Description** | **Task Instructions Given to DDYPG Member** |
| --- | --- | --- |
| **Task 1 – GPI Creative Work**  The aim of Task 1 was for young people in the DDYPG to make a creative piece to bring to the first workshop for discussion. Each piece was to be based on a Good Practice Indicator [1] that they had read and found personally resonated with them in some way. | Members were sent a welcome pack, this included creative materials: a small sketchbook, a ballpoint pen, a pencil, a marker pen, some mental health related and shape stickers, a glue pen, an A4 page with a template of a smartphone, a page from a book, and a copy of the Good Practice Indicators written as a list.  They were given instructions for two potential creative methods: erasure poems, and using a template for drawing. However, they were also encouraged to use their own preferred creative methods to produce something if they preferred.  **Erasure poems**: A page from a book, either ‘Do No Harm’ by Henry Marsh [3] or ‘It’s All in Your Head’ by Suzanne O’Sullivan [4] were sent to each member for the creation of an erasure (or blackout) poem. These books were selected because of their relevance to the medical field which offered relevant terminology for young people to creatively engage with. This method promotes critical thinking and personal reflection [5]  **Template for drawing:** A blank smartphone outline, sized to fit an A4 sheet of paper, was provided to each member as an art prompt. The template served as a guide to help young people engage with a familiar object associated with our research topic [6]. This method also encouraged members to explore their personal experiences and connections to their smartphones and devices.  Members received the welcome pack to a provided address with instructions for Task 1. | GPI Creative Work instructions were provided as follows:  You will find a copy of the ‘Good Practice Indicators’ (GPIs) included. Please give these a read.  We would like you to pick one of the GPIs that resonated with you the most (most relevant, most important, most overlooked), and we’d like you to portray your feelings around this in a creative way.  You can use your own artistic style, or one of those included in this pack. Please spend around 20 minutes on the task.  We will discuss the creations in our first session. If you’re happy to share please send your final creation (or a picture of it) to Zoe via email. She will remind you about this before the meeting if needed.  Thanks! |
| **Task 2: Survey**  The aim of Task 2 was to gather young people's perspectives on online culture, mental health, and digital communication through a short survey. The results were intended for discussion during Workshop 2, alongside data from: i) our research survey on mental health professionals' (MHPs) training needs for engaging with young people on these topics [2]  ii) additional information on development of the Good Practice Indicators [1]. | Members were provided with a link to the survey via email and Discord, hosted on Google Forms.  The survey explored various aspects of members’ online experiences. Questions about emojis, for example, aimed to understand how members interpreted different symbols and how these shaped their online communication. We also asked about the platforms they used to access mental health content, providing insight into the diversity of online spaces they engaged with. Questions about online trends helped us gauge their awareness of potentially harmful patterns, while asking about influencers allowed us to understand who shaped their mental health perceptions. The survey further inquired into their positive experiences with MHPs when discussing online use, as well as their expertise in internet slang and mental health terms to enrich our understanding of their digital behaviours. | Survey instructions were provided as follows:  We have created a short optional survey to inform our next workshop meeting. The data will be shared and discussed during the session.  Survey questions included: What does this emoji 💀 mean to you and when might you use it?  What does this emoji ⏳ mean to you and when might you use it?  What does this emoji 💅 mean to you and when might you use it?  What does this emoji 😭 mean to you and when might you use it?  What does this emoji 😂 mean to you and when might you use it?  What apps/platforms/sites have you used to access mental health content online? (please list all and be specific, including both past and current examples)  What online trends (around mental health) have you heard about or seen?  What influencers do you follow that talk about mental health? (In what ways do they talk about it?)  Have you ever had a good experience of talking to a mental health professional about your online use? (what went right?)  Teach me some internet slang/terminology (especially any related to mental health) please: |
| **Task 3 – Search History Poems**  The aim of Task 3 was for DDYPG members to create list poems reflecting on online searches they had made, exploring their connections to their mental health at the time. This method of poetic inquiry allowed members to delve into their personal experiences, thoughts, and actions related to their digital behaviours and mental health. | Task instructions were shared on Discord and emailed to members.  To inspire their work, we provided a list-style poem, "Amazon History of a Former Nail Salon Worker" by Ocean Vuong [7], which used online search histories to construct a reflective narrative. | Search History Poem instructions were provided as follows:  I want to introduce the next task for the DDYPG, which is focused on two poetry techniques. We will be using the two attached poems in the 'poetry examples' document as inspiration for our own work. This type of poetry uses ‘list poetry’ and ‘digital age’ techniques, where we take the seemingly mundane – such as online search histories – and use it to construct a narrative.  We want you to use examples of searches (fictionalised or real), to create a poem that expresses your online journey in relation to your mental health.  The period this covers is up to you, so it may start when you were at a stage of declining mental health and show the change to improved mental health, or it may be less linear and show the ups and downs in the road. It may only be this year, or it may be a year in the past, or based on multiple years. You may want to use real-life examples of terms you searched for, or instead use fake searches that accurately describe what you wanted to see/how you behaved during this time.  I have also included examples of poems for you to view. |
| **Task 4 – Character Development**  The aim of Task 4 was to begin developing a character for a video resource we intended to create. | During Workshop 3, a Digital Education Developer from the university presented an overview of his work and how we could apply it to our findings from Workshops 2 and 3 to develop a practical resource.  One suggestion was to create a video that portrays a ‘day in the life’ of someone engaging with mental health content online. The group was enthusiastic about the idea, particularly in highlighting how people’s mental health states can influence their interaction with online content. It was also suggested that this approach could offer a realistic alternative to the often idealised ‘day in the life’ videos commonly seen online.  To proceed, we agreed that developing character profiles would be the next step. The group was asked to complete a character development plan. | Character development instructions were provided as follows:  Please complete the following document and return it to Zoe:  **History**  Write a brief back story of your main character (we can expand on this later). Map where their issues with mental health began, up to the present day. Have they contacted MHPs before (they may not have)? What was that experience like?  **Social Media**  How do they use social media? What type of content is beneficial to them and what is problematic? Perhaps you could refer to real-life examples. Please write descriptively about their emotional response.  **Events**  Create some everyday experiences in your character's world (which would be reasonable to film) where the influence of social media impacts their decision-making and behaviour.  (e.g. they will watch videos of someone's 'unrealistic' day-in-the life where they do several activities including going to the gym, eating fruit for breakfast, cleaning, making a coffee, and getting work done all before 8am, this leaves my character feeling guilty, inadequate, and anxious, resulting in them staying in bed and scrolling on their phone meaning they miss eating dinner and don't go to sleep until 3am).  **MHP**  Your main character meets with a MHP. What happens during that meeting? Especially when the MHP asks about their online experiences and how their online use could be managed. Consider what would be helpful and not so helpful in terms of the practitioner's approach. How might this make the main character feel? What could make them feel supported, and what could make them feel judged? You may want to write two versions, with both positive and negative outcomes. |
